# Economic and health consequences of non-invasive respiratory support in newborn infants: a difference-in-difference analysis using data from the Norwegian patient registry

**DOI:** 10.1186/s12913-014-0494-4

**Published:** 2014-11-01

**Authors:** Inger Cathrine Kann, Anne Lee Solevåg

**Affiliations:** The Health Services Research Centre HØKH, Akershus University Hospital, Lørenskog, Norway; The Department of Pediatric and Adolescent Medicine, Akershus University Hospital, Lørenskog, Norway

## Abstract

**Background:**

Newborn infants with respiratory failure are often treated with intubation and mechanical ventilation for prolonged periods of time. Our objective was to evaluate whether increasing use of non-invasive respiratory support in newborn infants can improve patient health and reduce costs.

**Methods:**

We utilized a natural experiment that took place in October 2008 when a large neonatal intensive care unit in Norway moved into a new hospital building with new medical equipment. A change in respiratory support towards increasing use of nasal biphasic positive airway pressure (n-BiPAP) instead of invasive mechanical ventilation treatment followed the acquisition of the new equipment. We used a difference-in-difference method and data from the Norwegian National Patient Registry to assess morbidity, mortality, number of hospital days and hospital costs in our unit following this change. We stratified the results according to gestational age groups.

**Results:**

We found a reduction in morbidity including bronchopulmonary dysplasia, retinopathy of prematurity and intraventricular hemorrhage. No change in mortality was found. We found a reduction in number of hospital days and hospital costs for preterm infants with gestational age <28 weeks and for term infants with diagnoses affecting respiration.

**Conclusions:**

We conclude that increasing use of n-BiPAP may improve health and reduce costs. However, more research is needed to establish best practice. Comparing hospitals where treatment practices change to hospitals where the same change does not occur may be a useful way to evaluate the efficacy of such a change, especially when hospitals can be studied over time.

## Background

Newborn infants with respiratory failure are often treated with intubation and mechanical ventilation for prolonged periods of time. In this study, we evaluated health outcomes of patients and hospital costs associated with an increasing use of nasal biphasic positive airway pressure (n-BiPAP) instead of mechanical ventilation treatment as the mode of respiratory support in term and preterm infants with respiratory failure. We hypothesized that increasing the use of n-BiPAP reduced morbidity and mortality as well as the number of hospital days and costs.

In newborn infants, mechanical ventilation through an endotracheal tube is associated with both short- and long-term complications [1]. On the other hand, non-invasive respiratory support has been demonstrated to be less injurious [2]. Early nasal continuous positive airway pressure (n-CPAP) can reduce exposure to or duration of mechanical ventilation in extremely preterm infants [3] by, for instance, reducing the risk of extubation failure [4]. N-BiPAP is another mode of non-invasive respiratory support that may additionally reduce the need for mechanical ventilation [5,6] and complications like bronchopulmonary dysplasia (BPD), retinopathy of prematurity (ROP), intraventricular hemorrhage (IVH), periventricular leukomalacia (PVL) and necrotizing enterocolitis (NEC), which have been associated with mechanical ventilation treatment in preterm infants.

In October 2008 Akershus University Hospital (Ahus) in Norway moved into a new hospital building and the new neonatal intensive care unit (NICU) acquired new equipment for non-invasive respiratory support. These machines allow for both n-CPAP and n-BiPAP treatment and had not been used in more than a few other hospitals in Norway at the time. Based on emerging knowledge that n-BiPAP improved gas exchange compared to n-CPAP [7], we started to use n-BiPAP, primarily in preterm infants with inadequate oxygenation and/or ventilation on n-CPAP. We also used n-BiPAP in term or near-term infants with respiratory symptoms of any cause that experienced n-CPAP failure. We utilized a treatment protocol similar to those published for nasal intermittent positive pressure ventilation (NIPPV) with short inflation times of 0.3-0.4 s and high rates of 40–60 per minute. However, since our equipment only allows for maximum pressures of 9–10 cm H_2_O, we called this mode of respiratory support n-BiPAP, even though higher inflation times (or time high [Thigh]) and lower rates are more commonly used in this treatment modality. We assumed that other hospitals that did not utilize n-BiPAP would have to intubate and mechanically ventilate newborn infants that did not respond properly to n-CPAP treatment, whether n-CPAP was used as primary treatment or post-extubation.

To make causal inference, on what medical treatment is most efficient and improves health most, is a challenge. The most common method is randomized controlled trials (RCTs) that select patients randomly to different treatment methods. We instead utilized the change in treatment practices that took place when Ahus moved into a new hospital building and the change in respiratory support towards increasing use of n-BiPAP instead of invasive mechanical ventilation treatment that followed.

This change in treatment practices may be seen as a natural experiment. Such a quasi-experimental design has a long tradition in social sciences [8,9], but we argue that such methods could also be utilized in medicine where RCTs are the gold standard for comparing treatments, medical equipment, new technology or treatment modes.

In Norway, the provision of health care is organized through health enterprises that are owned and governed by the state regional health authorities. The regional health authorities are financed by a combination of block grants and an activity-based system. Together, the two types of funding are meant to cover the running costs of providing somatic hospital services. The government determines the relative shares of the two sources of funding on a yearly basis. The activity-based share is based on the Diagnosis-Related Group (DRG) system and accounted for 40–60% of the funding in the observation period. The DRG system is meant to reflect the average costs of providing treatment to patients within different DRGs [10]. One patient can be categorized to several combined DRGs, depending on treatment procedures. As DRG is based on an estimated cost of a certain patient group, reducing expenditures, by for instance reducing length of stay, for this patient group will improve hospital economy, at least in the short term.

Infants on mechanical ventilator treatment often require sedation and analgesia, arterial and central intravenous lines, antibiotics and parenteral nutrition. Mechanical ventilation may thus be expensive compared to n-BiPAP due to the need for more intensive monitoring and care. Increased use of n-BiPAP may therefore reduce hospital costs as well as the number of hospital days (NHD). These economic effects might be enhanced by reduced short- and long-term morbidity in patients receiving n-BiPAP instead of mechanical ventilation.

## Methods

Ideally, in order to capture the effects of a change in treatment procedures, we should know what the outcomes for the exposed patients would have been in the original treatment regime. Since this cannot be observed, the outcomes for patients treated in hospitals that did not use n-BiPAP represent the counterfactual.

We used a difference-in-difference (DID) approach [11]. Having data from 2002 to 2010, we were able to observe outcomes in exposed and unexposed hospitals before, and after, the change towards increasing use of n-BiPAP. This allows us to control for the possibility that both groups have changed over time for reasons unrelated to the intervention or that they were different in some aspects at baseline. In effect, the analysis isolates the impact of the intervention by removing other known or unknown factors that may have affected the groups during the study period.

The DID design is based on comparing four different groups. Three of these groups are not affected by the investigated treatment, which in this study was n-BiBAP. The difference between Ahus before November 1st 2008 (a) and after October 31st 2008 (b) compared to the difference between the unexposed hospitals before November 1st (c) and after October 31st 2008 (d) was calculated. The DID unadjusted is: (b-a)-(d-c). Thus, if the exposed and unexposed are subject to the same time trends, such as changes in medication practices like the use of antenatal steroids and surfactant treatment, this will not confound the results, since we use the change in the other hospitals as a reference.

No other changes in the treatment protocols of sick term and preterm infants took place at Ahus at the same time. There were no big changes in the care of the infants’ mothers prior to birth such as the use of antenatal steroids, other than the national trends. There has been a move towards less mechanical ventilation in newborns in general the last decade, unrelated to the use of n-BiPAP. However, since we in the DID approach use the trends in other hospitals as reference, such trends will not confound the results.

We performed regression analyses using individual data; adjusted for compositional differences in the patient populations by including gender, birth weight, and patent ductus arteriosus (PDA); and took into account whether or not the admission was planned. In Norway, a planned hospital admission of an infant the first year of life is usually due to a transfer for a procedure or treatment at a tertiary referral center and then back to the referring hospital. All other admissions the first year of life, including the admission to the NICU at birth, are defined as unplanned and may indicate differences in health status of the individuals included in the study.

All outcome parameters were analyzed within the DID framework. However, the outcome parameters were in different formats, and thus, different regressions were used. For the linear outcome parameters; NHD and hospital costs (DRGs), an ordinary least-square regression was used. However, these variables all demonstrated a skewed distribution. Thus, a log transformation was also performed. An ordinary least-square regression on log-transformed data has often been used in this kind of data and appears to be a consistently performing estimator [12]. Regarding the outcome of mortality, we had the date of death and thus a Cox regression was used to estimate the effect on mortality. Logistic regressions were used for the other health outcomes. Regression results are presented with 95% confidence intervals (CI). All analyses were conducted in Stata, version 12 (StataCorp LP, Texas, USA).

We used individual data from the Norwegian Patient Register (NPR) 2002–2010.

For each patient, we have information on NHD, gender, DRG, diagnoses, and treatment procedures, whether or not the admission was planned, in which hospital the treatment was performed and the date of death recorded if the patient died in the hospital. For all patients, we included diagnoses and treatment procedures per patient, per year and per hospital, regardless whether they were primary or secondary diagnoses or procedures. This is important, since what is reported as primary diagnoses may be a strategic choice of hospitals according to DRG rates [13].

The main outcome parameters for the preterm infants were morbidity, defined as BPD, ROP, IVH, PVL and/or NEC and in-hospital mortality; as well as NHD, defined as the number of hospital days per patient within the same year and hospital; and hospital costs within the same year and hospital, measured in aggregated DRGs per patient. For term infants, the outcomes were only NHD and hospital costs (DRGs).

We included only hospitals certified to care for preterm infants with gestational age (GA) <28 weeks, by which we attempted to reduce heterogeneity. We excluded other hospitals that started to utilize n-BiPAP during the observation period. These hospitals could have been included in the treatment (exposed) group. However, as indications for and extent of use of n-BiPAP varied among exposed hospitals, and the exact date when n-BiPAP was introduced as a treatment modality in these hospitals could not be established, we decided to exclude them from the main analysis.

We defined an infant as being born the same year he or she was admitted to the hospital. Our unit of observation was preterm infants as well as term infants with diagnoses affecting respiration by hospital and year. Diagnoses that frequently affect respiration and cause a need for respiratory support include ‘respiratory failure’, respiratory distress syndrome, persistent pulmonary hypertension, transitory tachypnoe of the newborn/wet lung, meconium aspiration syndrome and perinatal asphyxia.

**Figure 1:**
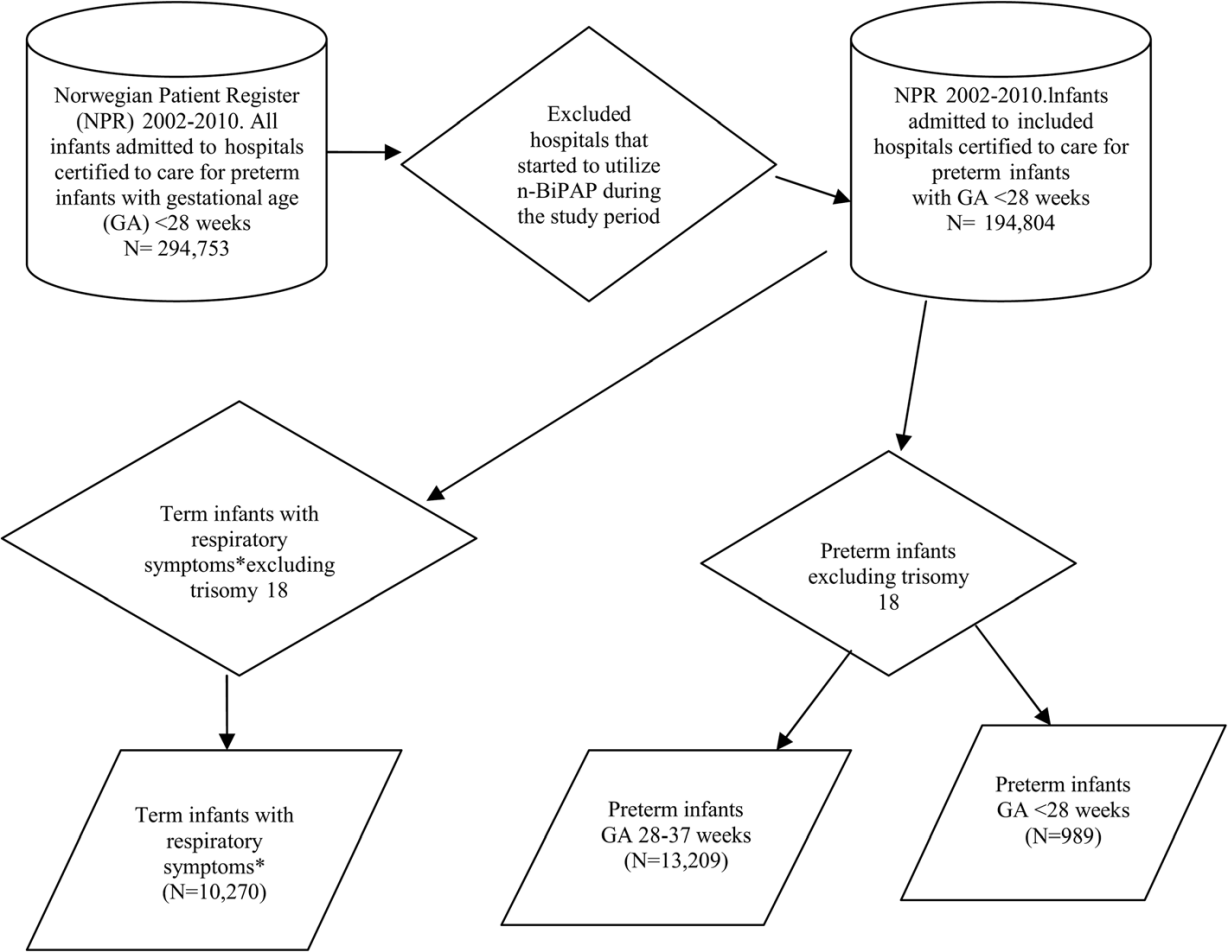
**Flow chart describing the study population.** *’Respiratory symptoms’ include the diagnoses respiratory failure, respiratory distress syndrome, persistent pulmonary hypertension, transitory tachypnoe of the newborn/wet lung, meconium aspiration syndrome and perinatal asphyxia.

The NPR assigned patients with a new identification (ID) number each year, and in each hospital, the patients were admitted before 2008. After 2008, the NPR allows for tracking individual patients between years and hospitals. In order to make data after 2008 comparable with those before 2008, we gave each patient a new ID for each year and hospital. We stratified the data according to the gestational age groups extremely preterm infants (GA <28 weeks), other preterm infants (GA ≥28 but <37 weeks), and term infants with respiratory symptoms (Figure 1).

By coincidence, three out of six infants who died at Ahus in 2010 had trisomy 18. Hence, we excluded all infants (13 preterm and 18 term infants) with this severe chromosomal abnormality that is associated with high mortality and morbidity but cannot be improved by the new treatment procedure.

In Norway, pregnant women can choose which hospital to give birth in, which in general is the hospital closest to their home. The exception is an anticipated very preterm birth which is regarded as an emergency, and the woman will deliver in the most nearby hospital, unless she is expected to give birth prior to gestational week 26. Caring for infants younger than 26 weeks of gestation is a centralized task, as is cardiac anomalies or other severe malformations diagnosed in the fetus. We allowed for an individual hospital effect by a dummy per hospital due to some of the resulting differences in the patient population between hospitals.

### Ethics statement

The research has been performed in accordance with the Declaration of Helsinki. We used data from the Norwegian patient registry (NPR). This is a registry including all patients treated in all Norwegian hospitals. The data were processed in accordance with guidelines issued by the Local Privacy Legislation Authority, and results are presented in a manner that preserves personal anonymity. Using data from NPR was approved by The Norwegian Data Protection Authority, reference- 11/00266-4/CGN. Individual consent was not required as we utilized data from NPR and the natural experiment that took place when Ahus moved into a new hospital buildings and the change in respiratory support that followed. All infants in all Norwegian hospitals were treated according to best practice and guidelines available at the time. This study is an assessment of minor differences in treatment practices. Such quality assessments were one of the political motivations for establishing NPR registry.

## Results

Table 1 compares the outcome variables, for the four groups Ahus before November 1st 2008 (a) and Ahus after October 31st 2008 (b) compared to the unexposed before November 1st 2008 (c) and after October 31st 2008 (d), stratified by the three gestational age groups. On average, treatment costs for extremely preterm infants (GA <28 weeks) at Ahus were reduced from 10.7 to 6.7 DRGs, while we observed a slight increase from 13.0 to 13.8 in other hospitals during the same period. The unadjusted DID is (6.7-10.7)-(13.8-13.0) = −4.7 DRGs. Thus, the change in Ahus is measured using the change in other hospitals as reference. A reduction by 4.7 DRGs, represents approximately 22,000 EUR per patient. The extremely preterm infants spent on average 47 days in hospital before Ahus moved into a new hospital building and 18 days after, while other hospitals changed from 37 to 34 days, i.e. an unadjusted effect of (47–18)-(37–34) =26 days. NHD in term infants with diagnoses affecting respiration changed from 8.4 to 4.9 days at Ahus and from 7.3 to 6.9 in the other hospitals, thus the unadjusted effect is 3.1 days for term infants with respiratory problems.

**Table 1:** **Descriptive statistics, before and after the introduction of n-BiPAP treatment at Ahus stratified by gestational age groups**

	**Ahus before November 1st 2008 (a)**	**Ahus after October 31st 2008 (b)**	**All hospitals but Ahus before November 1st 2008 (c)**	**All hospitals but Ahus after October 31st 2008 (d)**	**Difference in difference (b-a)-(d-c)**
**Preterm infants <28 weeks (N =989)**					
	Fraction	Fraction	Fraction	Fraction	(b-a)-(d-c)
BPD	0.313	0.094	0.243	0.116	−0.091
ROP	0.063	0.000	0.106	0.184	−0.141
IVH	0.063	0.038	0.244	0.168	0.051
Combined morbidity	0.375	0.151	0.502	0.421	−0.144
In-hospital death	0.107	0.075	0.268	0.221	0.015
	Mean	Mean	Mean	Mean	(b-a)-(d-c)
NHD	47.964	18.585	37.282	34.116	−26.213
DRG	10.729	6.709	13.066	13.842	−4.796
N	112	53	634	190	
**Preterm infants 28–37 weeks (N =13209)**					
	Fraction	Fraction	Fraction	Fraction	(b-a)-(d-c)
BPD	0.023	0.006	0.016	0.009	−0.010
ROP	0.004	0.006	0.013	0.025	−0.010
IVH	0.010	0.006	0.019	0.016	−0.001
Combined morbidity	0.032	0.017	0.044	0.046	−0.018
In hospital death	0.004	0.009	0.015	0.014	0.006
	Mean	Mean	Mean	Mean	(b-a)-(d-c)
NHD	17.246	16.494	15.573	15.175	−0.354
DRG	4.939	5.130	5.097	5.609	−0.321
N	1680	538	8290	2701	
**Term infants with respiratory symptoms (N =10270):**					
	Mean	Mean	Mean	Mean	(b-a)-(d-c)
NHD	8.433	4.913	7.332	6.916	−3.104
DRG	2.009	1.431	1.767	2.253	−1.064
N	1082	482	6646	2060	

Patent ductus arteriousus (*PDA*), Bronchopulmonary dysplasia (*BPD*), Retinopathy of prematurity (*ROP*), Intraventricular hemorrhage (*IVH*), Number of hospital days (*NHD*), Nasal biphasic positive airway pressure (*n-BiPAP*), Akershus University Hospital (*Ahus*).

Table 2 displays the results from the logistic regression and the Cox regression. We found a reduced morbidity by approximately 60% in the exposed population at Ahus for both extremely preterm and other preterm infants when adjusting for confounding variables, No change in mortality after the introduction of n-BiPAP treatment was found. Table 3 displays the results from the ordinary least-square regression. For the extremely preterm infants, we found a reduction in NHD by 70% at Ahus after October 31st 2008, or 26 days on average. Costs measured by DRG points were also reduced by 67% (Table 3). For this patient group, this represents (≈26 patients per year)*60.000 (average cost per patient)*48% ≈ 212,655 EUR (95% CI 107,092 EUR −1,483,995 EUR) saved per year at Ahus.

**Table 2:** **Estimated effect on health outcomes, stratified by gestational age**

	**Logistic regression**				**Cox regression**
	**Odds ratio**	**Odds ratio**	**Odds ratio**	**Odds ratio**	**Hasard ratio**
	**ROP**	**BPD**	**IVH**	**Morbidity**	**Mortality**
**Preterm infants <28 weeks (N =989)**					
DID-estimate	.	0.350***	0.674	0.393***	1.945
95% CI	.	[0.305,0.402]	[0.202,2.247]	[0.287,0.540]	[0.217,17.409]
**Preterm infants 28–37 weeks (N =13209)**					
DID-estimate	1.392	0.243***	0.397***	0.397***	2.143
95% CI	[0.747,2.595]	[0.200,0.295]	[0.321,0.492]	[0.337,0.468]	[0.335,13.697]

The results are adjusted for weight, gender, PDA and whether or not the admission was planned.

***p <0.001.

Bronchopulmonary dysplasia (*BPD*), Retinopathy of prematurity (ROP), Intraventricular hemorrhage (*IVH*), Difference-in-difference (*DID*), Confidence interval (*CI*). No observations of ROP at Ahus after the intervention, a change cannot be estimated.

**Table 3:** **Estimated effect on costs and number of hospital days**, **stratified by gestational age**

	**Ordinary least square regression**		**Ordinary least square regression**	
	**Log transformed**			
	**Number of hospital days**	**DRG points**	**Number of hospital days**	**DRG points**
**Preterm infants <28 weeks (N =989):**				
DID-estimate	−0.861*	−0.482*	−26.60*	−5.428***
95% CI	[−1.565,-0.157]	[−0.931,-0.0326]	[−47.46,-5.738]	[−8.344,-2.513]
N	989	989	989	989
R-sq	0.076	0.095	0.076	0.115
**Preterm infants 28–37 weeks (N =13209):**				
DID-estimate	−0.05	0.017	−1.656	−0.266
95% CI	[−0.242,0.142]	[−0.078,0.113]	[−4.839,1.527]	[−0.787,0.254]
N	13209	13209	13209	13209
R-sq	0.322	0.206	0.327	0.323
**Term infants - with respiratory problems (N =10270):**				
DID-estimate	−0.257**	−0.326***	−3.023**	−1.288***
	[−0.443,-0.0705]	[−0.443,-0.209]	[−5.113,-0.933]	[−1.897,-0.680]
N	10270	10270	10270	10270
*R-sq*	0.07	0.172	0.078	0.21

The results are adjusted for weight, gender, patent ductus arteriosus and whether or not the admission was planned.

*p <0.05, **p <0.01, ***p <0.001, confidence intervals in brackets.

Difference-in-difference (*DID*), Confidence interval (*CI*).

For term infants, we found a reduction in NHD days by 75%, or 1–5 days. The costs measured by DRG points were reduced by 67% or by 0.6 to 1.8 points. For this patient group, this represents (≈250 patients per year)*9.000 (average cost per patient)*33% ≈ 1,521,524 EUR (95% CI 1,264,175 EUR - 1,805,964 EUR) saved per year at Ahus. We found no change in NHD or DRG for other preterm infants.

Complications of prematurity and other complications in the perinatal period can lead to major disability like cerebral palsy in the survivors. The long-term costs of patients with, for instance, BPD, ROP and cerebral palsy resulting from IVH can be great. However, as there is no precise way to quantify such costs, we chose to extrapolate by using compensations received by plaintiffs that have suffered from medical malpractice in the perinatal period. In Norway, such reimbursement for cerebral palsy caused by IVH has in recent years been approximately 400,000 EUR per child with a severe disability. Severe ROP was reimbursed by 1,000,000 EUR in one severe case. Having one or more of the morbidities and assuming a reduction by ≈ 60% (Table 2), this represents approximately 10 patients per year at Ahus, i.e., 2,400,000-6,000,000 EUR saved in costs associated with long-term disability at Ahus per year.

The cost related to a change in mode of treatment similar to the one made at Ahus are only investment costs, since costs associated with the education of staff in the new mode of assisted ventilation are minimal. All hospitals will still need equipment related to mechanical ventilation assistance, i.e., business as usual will have no change in investment. Ahus has purchased nine machines, which the staff evaluated as a sufficient number. If we assume that they can be used in 10 years, the yearly cost for Ahus is approximately 100,000 EUR per year. Accordingly, the savings far exceed the costs, even at the lowest estimates of savings.

## Discussion

In this study, we found that morbidity and number of hospital days and hospital costs were significantly reduced following a change toward an increasing use of non-invasive respiratory support for preterm infants. For term infants, we found a reduction in NHD and hospital costs. The need for invasive mechanical ventilation may have been reduced, as well as the need for staff due to easier surveillance and shorter hospital stays in the initial treatment of the newborn infant.

Using changes in treatment practices or investments in one hospital that does not happen in other hospitals at the same time may be a way of evaluating procedural and technological changes as long as we have data on outcomes both before and after the change for both the exposed and unexposed hospitals. In this way, we can estimate opportunities for improved health, reduced costs, and/or increased efficiency while the changes are used in a normal hospital setting.

Some patients may have a prolonged stay in local hospitals following discharge from the study hospitals. However, Ahus only occasionally discharges newborns to other NICUs and almost invariably discharges infants to their home. NHD at Ahus therefore most often reflects the total length of hospital stay in the neonatal period, and the reduction of NHD at Ahus after October 31st 2008 is not likely to be associated with prolonged stays in other units. However, the NPR does not have information about this, since it does not allow for following patients between hospitals over the entire study period. In Norway, delivery and treatment of extremely premature infants are centralized tasks. Once infants are stabilized, often synonymous with being off mechanical ventilation, they are transferred to hospitals closer to home. Hence, in general, a reduction in ventilator days would allow for an earlier transfer to local hospitals and additional benefits for patients, neonatal service provision and for families.

The regression does not indicate changed mortality, though the estimates are based on few observations. In the patient group with highest mortality, i.e. the patients born before 28 weeks of gestation, no increase in mortality was seen. However, early mortality with a non-invasive treatment method needs more research and should be followed closely.

Using large population registers for evaluating changes in treatment procedures that occur in one unit is important regarding the costs associated with registry administration. Many countries cannot afford managing such a total patient registry. Norway has chosen to prioritize gathering data on all patients admitted to hospital since 1997. It is important to use such registers in research also in order to improve the quality of registration and coding practices.

Limitations of the study include its retrospective nature. Changes other than n-BiPAP may have occurred at Ahus only and at the time when Ahus moved into a new hospital building. Even though we controlled for some confounding variables, we cannot conclude that the observed changes were due to the new treatment procedures under investigation. Moving into new hospital buildings may have affected our patient group in ways for which we did not account. Renewal of all medical equipment, besides the n-CPAP/n-BiPAP machines, is likely to have improved efficiency and quality of care. However, the move was not associated with significant changes in staff or other medical guidelines.

Thus to check the robustness of the results, we performed the regression including the other exposed hospitals that we excluded from our main analyses. In this analysis we excluded Ahus. With this alternative regression, we found similar effects on morbidity and hospital costs, and most health outcomes for extremely preterm infants. This strengthens our results. However, the alternative regression indicated increased mortality among the smallest babies. The protocols for n-BiPAP usage in different units may play a role in mortality, and further studies are required. We did not include these units in the main study, because we only knew the month of purchase of the equipment that allowed for n-BiPAP treatment. We did not know if, when or on what indications the n-BiPAP treatment was used. However, including these units has an advantage, since the different units bought the equipment at different times, which makes the regression results more robust.

The NPR did not allow us to follow individual patients as they were transferred between hospitals and units. This makes us unable to make conclusions about the total length of hospital stay for each infant. However, the NHD in our unit was significantly reduced after we started to treat infants with n-BiPAP. As our unit is not a tertiary referral center, we almost exclusively treat infants that belong to our own referral area. As previously mentioned, very few patients are transferred to other units after discharge from our NICU. Hence, the possibility that a shorter stay in our NICU is followed by a longer stay in another unit is unlikely.

There has been a move towards less mechanical ventilation in newborns in general the last decade, unrelated to the use of n-BiPAP. However, the DID approach will ensure that this will not confound the results. It is likely that Ahus has followed national guidelines concerning these treatments. If the levels of antenatal steroids and surfactant treatment, are different in various hospitals, or if they change over time, this will not confound the results. This is resolved using the DID approach.

There has been no other major change in the care of preterm infants the last decade comparable to antenatal steroids and surfactant treatment of respiratory distress syndrome 20 years ago. However, if there had been changes in such use, it would only confound our results if it happened at Ahus only, and at the same time as Ahus moved into a new hospital building.

## Conclusions

In conclusion, assessing the effect of a shift towards n-BiPAP instead of mechanical ventilation treatment of term and preterm infants in need of respiratory support, by using a population register, we found a reduction in morbidity, the number of hospital days needed, and hospital costs. The retrospective nature of the study offers challenges but also advantages. In prospective studies, reporting side effects and negative outcomes may be influenced by the staff being aware that the treatment practices are under evaluation, and we argue that population registers and DID methods can be useful in estimating effects of changes in treatment practices. A total population registry has the potential of evaluating the health effects and costs of investments within a DID framework, given that the investments are not introduced in all hospitals at the same time.

## Abbreviations

PDA: Patent ductus arteriosus
BPD: Bronchopulmonary dysplasia
ROP: Retinopathy of suprematurity
PVL: Periventricular leukomalacia
IVH: Intraventricular hemorrhage
NEC: Necrotizing enterocolitis
DID: Difference-in-difference
PDA: Patent ductus arteriosus
Ahus: Akershus University Hospital
n-BiPAP: Nasal biphasic positive airway pressure
n-CPAP: Nasal continuous positive airway pressure
